# A Systematic Review of Clinical Diagnostic Systems Used in the Diagnosis of Tuberculosis in Children

**DOI:** 10.1155/2012/401896

**Published:** 2012-07-17

**Authors:** Emily C. Pearce, Jason F. Woodward, Winstone M. Nyandiko, Rachel C. Vreeman, Samuel O. Ayaya

**Affiliations:** ^1^Department of Pediatrics, Indiana University School of Medicine, Indianapolis, IN 46202, USA; ^2^Department of Pediatrics, Moi University School of Medicine, Eldoret 30100, Kenya

## Abstract

*Background*. Tuberculosis (TB) is difficult to diagnose in children due to lack of a gold standard, especially in resource-limited settings. Scoring systems and diagnostic criteria are often used to assist in diagnosis; however their validity, especially in areas with high HIV prevalence, remains unclear. *Methods*. We searched online bibliographic databases, including MEDLINE and EMBASE. We selected all studies involving scoring systems or diagnostic criteria used to aid in the diagnosis of tuberculosis in children and extracted data from these studies. *Results*. The search yielded 2261 titles, of which 40 met selection criteria. Eighteen studies used point-based scoring systems. Eighteen studies used diagnostic criteria. Validation of these scoring systems yielded varying sensitivities as gold standards used ranged widely. Four studies evaluated and compared multiple scoring criteria. Ten studies selected for pulmonary tuberculosis. Five studies specifically evaluated the use of scoring systems in HIV-positive children, generally finding the specificity to be lower. *Conclusions*. Though scoring systems and diagnostic criteria remain widely used in the diagnosis of tuberculosis in children, validation has been difficult due to lack of an established and accessible gold standard. Estimates of sensitivity and specificity vary widely, especially in populations with high HIV co-infection.

## 1. Background

Tuberculosis (TB) remains one of the most important causes of pediatric mortality worldwide, especially in areas with high HIV prevalence. There are approximately nine million new TB cases each year, with ten percent of those occurring in children, equaling almost one million new pediatric cases each year. Seventy-five percent of those are in twenty-two high-burden countries, which also tend to have fewer resources for diagnosis. Accurate and timely diagnosis of pediatric TB remains crucial because children are more likely than adults to progress from latent infection to active TB disease [1].

One of the largest challenges in preventing morbidity and mortality from TB among the pediatric population is the difficulty in making a timely diagnosis. Diagnostic approaches relying on symptoms, chest radiographs, tuberculin skin tests, or cultures all have particular challenges within the pediatric population. TB symptoms vary and overlap with other common pediatric diseases, especially in children who are coinfected with TB and HIV. Cough, anorexia, and weight loss are common in TB but nonspecific and might lead to overdiagnosis if used alone [2].

Chest radiography also is difficult to interpret in pediatric patients, who are less likely to have cavitations or clear radiological signs of TB. Mediastinal lymphadenopathy is often regarded as a radiologic hallmark of primary TB; however, this is difficult to diagnose on a plain chest X-ray (CXR), which may be of variable quality, particularly in some resource-limited settings. Also, significant interobserver variation exists when interpreting pediatric CXR for TB diagnosis [3].

Previous studies have shown various utility in using the tuberculin skin test (TST) in a highly BCG vaccinated population due to a concern for a high rate of false positives [4]. Though some evidence has shown that BCG-vaccinated children with known exposure to TB have a higher rate of positive tests than community controls [5], this study did not address the utility in other populations where TST may not be as sensitive, such as HIV-infected or malnourished children.

Pediatric TB tends to be pauci-bacillary and thus it is also more difficult to diagnose using cultures, especially in children who are too young to provide sputum [1]. Attempts have been made to improve the utility of culture-proven diagnosis by using induced sputum samples or gastric aspirates. These samples can still be difficult to obtain in children. Moreover, conducting these procedures in resource-limited settings can be difficult [6]. Because of the challenges in diagnosing pediatric TB through individual clinical signs and symptoms, radiological studies, or laboratory examinations, point-based scoring systems or diagnostic criteria are often used to assist in the diagnosis of TB in children.

The first major point-based scoring system was introduced by Stegen et al. in Chile in 1969 [7] and has continued to be modified and used around the world through the present [8–14]. The Keith Edwards criteria were originally published in 1987 [15] and also have been widely used [16–19] outside the original location of Papua New Guinea. Of the many diagnostic systems developed, the World Health Organization (WHO) criteria, originally published in 1983, are the most widely used [20]. The major objective of all of the diagnostic systems is to provide a consistent and accurate way to diagnose pediatric TB, especially in resource-limited settings.

Although these scoring systems and diagnostic criteria are commonly used [21], their reliability and validity remain unclear. Different diagnostic criteria are used in different settings, and they may or may not have been validated for those locations. Moreover, the challenges of using these criteria in settings where many of the children are malnourished or coinfected with HIV have not been fully examined. Many of the diagnostic systems were developed prior to the onset of the HIV epidemic and may not perform adequately in children with coinfection. Since TB is a leading cause of mortality among the world's 2.3 million HIV-infected children, diagnosing TB among coinfected children is a particularly important challenge and may require significant adaptations of current diagnostic systems [22].

Prevention of childhood morbidity and mortality due to TB requires accurate and timely diagnosis. A previous systematic review of pediatric TB diagnostic strategies, published in 2002, recommended standardization of definitions and characteristics, pointing out the need for new diagnostic approaches [21]. Since that review, at least twenty-one new papers on pediatric TB diagnosis have been published, including several highlighting new strategies such as the Brazil Ministry of Health system [23–25] and the Marais criteria [26]. In addition, the population of children living with HIV infection has reached 2.3 million, simultaneously expanding the numbers of children vulnerable to TB disease [22]. This systematic review seeks to systematically identify, review, and compare various methods of diagnosis of TB in children in order to inform clinical practice and future research in this area. It aims to organize the scoring systems and diagnostic criteria based on their common components, critically analyze the extent to which the criteria are validated, and highlight those that have focused specifically on children that are coinfected with HIV and TB.

## 2. Methods

We searched several bibliographic databases, including MEDLINE (through October 19, 2009), EMBASE, and relevant websites such as those for the World Health Organization. We used the following strategy: (tuberculosis/diagnosis) [MeSH heading] AND (criteria* OR screen* OR guideline* OR scor*). Three authors (S. O. Ayaya, J. F. Woodward, and E. C. Pearce) reviewed all returned titles and excluded articles that obviously did not involve children or tuberculosis. These authors then reviewed abstracts of remaining articles to determine which studies examined scoring systems or diagnostic criteria used in the diagnosis of pediatric tuberculosis. The bibliographies of all relevant articles were also reviewed for potential articles.

Two investigators (J. F. Woodward and E. C. Pearce) independently reviewed the remaining articles, independently deciding on inclusion in the review using a standard form with predetermined eligibility criteria. Disagreements were resolved by consensus. For inclusion, the articles needed to describe a descriptive or interventional study involving the use of a clinical diagnostic system to diagnose tuberculosis in pediatric patients. Only English language articles were included. Pediatric patients were described as individuals less than 18 years of age. Clinical diagnostic systems included both scoring systems and diagnostic criteria. Scoring systems were defined as point-based criteria with set numerical cutoffs for a positive diagnosis. Diagnostic criteria were defined as nonpoint-based systems in which a certain number of criteria out of the total or out of each group were needed for diagnosis. Studies analyzing the diagnosis of pediatric tuberculosis in general without using or evaluating a particular scoring system or diagnostic criteria were used as background information only for the review. Each article was analyzed to determine the study setting, study design and methods, sample characteristics, type of diagnostic system used, reference or gold standard used for comparison, and efforts at validation of the diagnostic system. We excluded duplicate publications of the same findings.

## 3. Results

The systematic literature search identified 2261 articles. The online search of MEDLINE yielded 2011 articles, and the search of EMBASE yielded 250 articles, many of which were also found by the MEDLINE search. Additional potential studies were identified through searches of bibliographies. After articles that did not address the diagnosis of tuberculosis in children were excluded, 408 articles remained. Further articles were excluded upon closer review because they did not include pediatric patients, did not include a scoring system or diagnostic criteria, or focused only on screening for latent tuberculosis. Articles that briefly mentioned a scoring system but did not give details or include how it was used in the study were also excluded. Forty articles met the general study criteria.

### 3.1. Clinical Diagnostic Systems Used for TB Diagnosis

From the forty articles that included a clinical diagnostic system, we extracted information on the setting, location, sample size, type of system/criteria used, efforts at validation, choice of gold standard, and the effect of HIV coinfection in the population. The characteristics of these studies, including the validation strategies, are summarized in Tables 1, 2, and 3. Eighteen studies used scoring systems; these studies could be further divided into five groups based on a common initial system modified by different authors (Table 1). The three major groups were the following: (1) the Kenneth Jones/Stegen-Toledo system [7–14]; (2) the Keith Edwards system [15–19]; (3) the Brazil Ministry of Health (MOH) system [23–25]. Fourie et al. [27] and Bergman [28] also presented new systems without further published studies. Eighteen studies used diagnostic criteria. These studies could be further divided into five groups of diagnostic criteria presented by Ghidey and Habte [29], Migliori et al. [30], Mahdi et al. [31], Salazar et al. [32], Marais et al. [26], the WHO guidelines [33–42], Osborne [43], Jeena et al. [44], and Ramachandran [45] (Table 2). Four articles compared two or more scoring criteria [46–49] (Table 3).

### 3.2. Validation of Clinical Diagnostic Systems for Pediatric TB Diagnosis

Of the above forty articles, sixteen attempted to validate the diagnostic system or systems (Table 4). Gold standards used in validation varied greatly and ranged from positive cultures to clinical diagnosis to previous scoring criteria. The only study using cultures as the primary gold standard was Sant'Anna et al. [24], which found a sensitivity of 89% and specificity of 86% when evaluating Brazil Ministry of Health criteria against a standard of culture-positive patients. Sant'Anna et al. [25] also performed a retrospective analysis on a different study population using clinical consensus as the gold standard against which to compare the diagnostic criteria, resulting in similar sensitivity.

Culture for *Mycobacterium tuberculosis *is less sensitive in pediatric patients and difficult to obtain in resource-limited settings; therefore, the most common gold standard used to validate diagnostic systems was clinical diagnosis. The definition of clinical diagnosis varied widely between studies and was often not defined in detail. Because many of the studies were retrospective, clinical diagnosis was often simply defined as children who had been admitted with a diagnosis of TB [8, 34], with some studies also specifying that the children must have improved on anti-TB medication [13, 40, 41]. In one article, the study population was drawn from forty-four different hospitals, all of which used their own methods of clinical diagnosis [17]. However, in other studies, the method of clinical diagnosis was explained in depth. For example, van Rheenen described a detailed algorithm that included clinical findings, culture, CXR, TST, contact history, and response to treatment [18].

Previously described scoring criteria were also used as a gold standard; a few of the studies compared their modifications of a certain diagnostic system to the original. For example, Migliori et al. modify the criteria published by Ghidey and Habte [29] by focusing the criteria on pulmonary TB and adding response to treatment and use the original criteria as the gold standard in their analysis [30]. Salazar et al. then modified the Migliori criteria to develop the Peru criteria, and used the original Migliori criteria as the gold standard for comparison [32]. These are not traditional validation strategies as they assume the previous criteria have been validated to an extent that they may now be considered a gold standard in themselves.

Four published papers evaluated and compared multiple scoring systems and diagnostic criteria (Table 3). In a 2002 systematic review, Hesseling et al. stressed the need for standardization of definitions and point values between the various algorithms [21]. As an update on Hesseling's review, this current review includes twenty-one new studies, including those evaluating the Brazil MOH scoring system [23–25] and the Marais criteria [26]. In 2008, Ahmed et al. published a review of TB diagnosis as well as treatment, focusing mainly on the Kenneth Jones and Keith Edwards systems [48] and suggesting the need for further research. Most recently, in 2009, Raquib et al. compared a newer diagnostic test (ALS assay) to clinical diagnosis, the Kenneth Jones, and the Keith Edwards scoring criteria [49], finding that sensitivity, specificity, and overall concordance was higher when the ALS assay was compared to clinical diagnosis than to the scoring criteria.

In a 2007 article, Edwards et al. used data from a retrospective review of TB cases at a pediatric hospital with a highprevalence of HIV infection to calculate scores for eight diagnostic scoring systems [47]. The decision to initiate treatment for TB was dependent on the scoring system used, with at least one scoring system recommending not to treat for 14% of the children studied. Except for the systems derived from a common original diagnostic system, correlation was poor to moderate for agreement of when to initiate treatment based on the various scoring systems.

### 3.3. Variation among Criteria

Although all of the scoring criteria have aspects in common, their purposes and specifics have varied over the past 40 years since Stegen et al. published the original Kenneth Jones criteria. The Kenneth Jones criteria include laboratory tests but exclude clinical criteria such as cough and fever due to concerns that they would lower the specificity [7]. In contrast, the purpose of the Keith Edwards criteria was focused towards a completely clinical diagnosis, and thus excluded laboratory data except for a TST [15].

Both the Kenneth Jones and Keith Edwards criteria were designed for the diagnosis of both pulmonary and extrapulmonary tuberculosis. Because the clinical signs and symptoms of extrapulmonary tuberculosis may differ from those of pulmonary tuberculosis, several studies evaluated the ability of diagnostic strategies to identify pulmonary TB specifically (Table 5). For example, the Brazil MOH system, designed specifically for pulmonary tuberculosis [24, 25], has shown a sensitivity of 89% and a specificity of 86%. The Migliori [30] and Marais [26] diagnostic criteria, also focused on pulmonary tuberculosis, demonstrated a sensitivity of 92% [31] and 82%, respectively. While the Migliori criteria have not been tested in children with coinfection, the sensitivity of the Marais criteria decreased to 51–56% when children under three years of age and HIV infected children were included [26].

A salient difference between the various clinical diagnostic approaches was the choice of included criteria. The criteria included most commonly were the tuberculin skin test (TST) and positive history of TB contact; however, the definition of these criteria was not standardized. For example, the definition of a positive TST varies widely among studies [7, 15]. A positive history of TB contact also was defined in various ways, such as requiring confirmed sputum-positive contact [37] or only a self-report of contact [30]. In some cases, the history of contact had to be within the past two years [14]. Using both the TST and the positive contact history may also be redundant if both are included. Variability is also seen in the other criteria, such as clinical symptoms and CXR. The various definitions and subjectivity of many of the criteria included in the diagnostic approaches make it difficult to compare the diagnostic strategies and the attempts at validation. In addition, clinicians likely vary in how they implement the scoring criteria, thus, making the diagnostic thresholds even less consistent.

### 3.4. Clinical Diagnostic Systems in HIV-Infected Patients

A few studies specifically examined TB diagnosis in HIV-infected children (Table 6). In his comparison of eight diagnostic scoring systems, Edwards showed that HIV-infected children tended to have higher scores, especially when the Keith Edwards system was used, leading to a concern for over-diagnosis of TB in HIV-infected children [47]. Marais et al. found that the Marais diagnostic criteria were less sensitive (56% compared to 82%) and less specific (62% compared to 90%) when evaluating children with HIV as opposed to children without HIV. The positive predictive value also decreased to 62% in HIV-infected children as compared to 82% in children without HIV [26]. Viani et al. looked at a small cohort of coinfected children in Mexico retrospectively and found that 77% had scores indicating highly probable TB when using the Stegen-Toledo criteria [8]. Finally, in a 2009 analysis of the Brazil MOH criteria, Pedrozo et al. found that while coinfected children did score slightly lower than HIV-uninfected children, their scores were still significantly higher than children without TB [25].

## 4. Discussion

We identified and reviewed forty different studies of twenty-two unique scoring systems or diagnostic criteria that were developed from five original scoring systems and five original diagnostic criteria. These diagnostic approaches varied in the types of clinical signs and symptoms included in the criteria, the inclusion or exclusion of laboratory testing, and even their diagnostic focus (i.e., pulmonary TB alone or pulmonary and extrapulmonary TB). Studies designed to validate the various diagnostic systems varied significantly in the gold standard chosen for comparison. Because the publication dates of the articles range over the last fifty years, some criteria were developed and evaluated prior to the HIV epidemic, while other studies focused specifically on coinfected children.

The gold standards chosen to evaluate the validity of these diagnostic strategies also varied widely. Cultures can be difficult to obtain in children. Because tuberculous disease in children is often pauci-bacillary, the diagnostic yield of cultures in children is often poor. Although one study used culture as the gold standard [25], others used positive response to treatment [13], CXR [35], or a previous scoring criteria [30]. The most common gold standard was clinical diagnosis. Interestingly, in a study of the ALS assay for diagnosing active TB disease, the assay actually correlated better with clinical diagnosis than either the Kenneth Jones or Keith Edwards scoring criteria [49]. Unfortunately, clinical diagnosis is likely to depend strongly upon the experience and knowledge base of the clinician and thus may be less reliable in settings where clinicians have less training. To allow for comparison of criteria across different studies and settings, future studies need to employ a more consistent gold standard. Ideally, this would be culture-based, as this is a standard for validation that could be reliably replicated across settings. However, because cultures are difficult to obtain in resource-limited settings and can lead to a delay in treatment, performing studies with culture as the gold standard can be difficult.

In addition to using a variety of gold standards, the various studies often included very different sample populations. Some studies did not clearly describe the characteristics of the patient population or how they were selected. Many were retrospective, often utilizing chart review. Ideally, prospective studies of diagnostic systems would evaluate a clearly defined sample of participants with a spectrum of disease that is representative of the patients to which the criteria would be applied in clinical practice. It is essential that researchers clearly describe the sample selection process and inclusion criteria in such studies to allow for more accurate comparisons of criteria across different populations or settings and to promote the utility of these systems in clinical practice.

Another challenge in prospective studies of TB diagnosis is the bias that is introduced when, as found in some of these studies, the inclusion or screening criteria for participants often include similar clinical features as the diagnostics systems being evaluated. For example, Pedrozo et al. used history of contact, CXR, and TST result as part of the criteria for inclusion in the study. Chest X-ray and TST were also used as part of their diagnostic gold standard to differentiate latent TB from no TB from active TB disease. All three inclusion criteria are also used in the Brazil MOH scoring system being evaluated in this study [25]. This makes it difficult to interpret the accuracy of a diagnostic system and its ability to predict a diagnosis of TB in a particular patient or patient population. This overlap also causes difficulty in determining the relative importance of particular signs or symptoms within the diagnostic system.

The largest shift in the newer diagnostic systems as compared to Kenneth Jones and Keith Edwards is the focus on pulmonary tuberculosis alone. Diagnostic systems focusing simply on pulmonary TB, such as the Brazil MOH and Marais criteria, have demonstrated higher sensitivities and specificities than those developed to diagnose both extrapulmonary and pulmonary TB. Because children have a higher incidence of extrapulmonary TB [50], using diagnostic systems targeted at pulmonary TB only addresses part of the diagnostic challenge. On the other hand, because TB presents with varied signs and symptoms depending on the site of disease, it is difficult to conceive of a single diagnostic system that could diagnose with high sensitivity and specificity the various types of tuberculosis infections (e.g., vertebral, abdominal, and pulmonary TB). Furthermore, many children with extrapulmonary TB also have pulmonary disease [51]. A new system of classification, focusing on the severity of the disease rather than location, has recently been published and may also be a more reliable and reproducible method. If this is well validated in different settings, it may allow various diagnostic systems to be better compared than is currently possible [52].

At this time, the Brazil MOH scoring system has the most studies evaluating its validity with consistently high sensitivities and specificities. In each of the three studies of this criteria, the scoring system was tested against a slightly different gold standard, ranging from clinical criteria [23, 25] to culture-proven disease [24]. Although this may make some comparisons difficult with the lack of a standard gold standard, the fact that the scoring system holds up fairly well when tested in different ways actually strengthens the evidence for its validity. Though it has not been tested outside of Brazil, it has been tested in both an inpatient [24] and outpatient setting [23, 25]. The performance of the scoring system has also been evaluated in HIV-infected patients. These coinfected children still scored significantly above the cutoff for a diagnosis of TB [23]. All of these evaluations point favorably toward the validity of this scoring system. Evaluating the Brazil MOH scoring system in additional settings worldwide should be an important next step.

The findings of this systematic review are limited by the design and quality of the studies included. The lack of consistent and sometimes clearly defined inclusion criteria among the studies makes it difficult to compare sensitivity and specificity across the different diagnostic systems. Most of the various diagnostic systems have only been evaluated in specific geographic locations or single populations; few studies evaluate a particular diagnostic system in multiple geographic regions or patient populations. Fewer studies have compared the diagnostic yield of multiple criteria in the same patient population. Finally, the increase in the prevalence of HIV during the publication range of these studies makes it difficult to compare studies from thirty years ago to those more recently published. Although this paper includes more than twenty new studies since Hesseling et al. was published in 2002 [21], the number of articles assessing the validity of each diagnostic system is still relatively small. The paper also did not include unpublished data or non-English publications.

## 5. Conclusion

Clinical diagnostic systems in use for many years (e.g., the original Kenneth Jones criteria) and those more recently developed (e.g., the Brazil MOH criteria) have generally been developed, and subsequently adapted, in an attempt to accurately and reliably diagnose tuberculosis in children. As more continues to be learned about the disease and newer, more accurate tests are developed, methods of diagnosis will likely be altered further. It remains crucial that these methods remain applicable to resource-limited settings where the majority of children with TB are still most likely to be found. Although the studies included in this paper are heterogeneous and difficult to compare, the Brazil MOH criteria seems to emerge as the best validated in children with TB alone as well as those coinfected with TB and HIV. Due to the difficulty with obtaining cultures and the expense of the newer diagnostic tests, clinical scoring systems and diagnostic criteria will likely continue to be necessary in resource-limited settings for some time. However, unless additional studies identify refined diagnostic systems with improved sensitivity and specificity, they will likely mainly be utilized as initial screening tools or adjuncts to support clinical diagnosis. Improving the accuracy of diagnosis of pediatric TB is needed to ensure appropriate and timely treatment of those with active disease and to prevent unnecessary morbidity and mortality. Validated clinical diagnostic systems that can be implemented in resource limited settings can improve the accuracy and timeliness of tuberculosis in children; however, additional well-designed studies are needed to validate the accuracy and reliability of current scoring systems and diagnostic criteria.

## Figures and Tables

**Table 1:** Point-based scoring systems and studies evaluating these systems.

Author	Year	Country	Scoring criteria	Changes	Study type
Stegen et al. [7]	1969	Chile	Kenneth Jones	New	Review with case reports
Mathur et al. [9]	1974	India	Kenneth Jones	Added marasmus to original criteria	Prospective observational
Nair and Philip [10]	1981	India	Kenneth Jones	Changed point values, took away negative points for BCG, added response to treatment	Prospective
Seth [11]	1991	India	Kenneth Jones	Used Nair's adaptation	Book excerpt
Shah et al. [12]	1992	India	Kenneth Jones	Added history of measles/whooping cough	Prospective observational
Mehnaz and Arif [13]	2005	Pakistan	Kenneth Jones	Modified multiple criteria, added and subtracted criteria	Retrospective case control
Oberhelmen et al. [14]	2006	Peru	Stegen-Toledo	No modifications	Prospective observational
Viani et al. [8]	2008	Mexico	Stegen-Toledo	Added points for positive stain	Retrospective chart review

Edwards [15]	1987	Papau New Guinea	Keith Edwards	Original	Review article
van Beekhuizen [16]	1998	Papua New Guinea	Keith Edwards	No modifications	Prospective observational
Weismuller et al. [17]	2002	Malawi	WHO score chart (modified Keith Edwards)	Added no response to malaria treatment, modified language	Cross-sectional observational study
van Rheenen [18]	2002	Zambia	Keith Edwards	Modified language	Prospective cohort
Narayan et al. [19]	2003	India	Keith Edwards	Added no response to malaria treatment	Prospective observational

Sant'Anna et al. [24]	2006	Brazil	Brazil Ministry of Health	New	Retrospective case control
Sant'Anna et al. [25]	2004	Brazil	Brazil Ministry of Health	No modifications	Retrospective
Pedrozo et al. [23]	2009	Brazil	Brazil Ministry of Health	No modifications	Prospective observational

Fourie et al. [27]	1998	Multiple	New	Set up new scoring criteria by consensus decision	Retrospective

Bergman [28]	1995	Zimbabwe	New	New	Review

**Table 2:** Diagnostic classifications and studies evaluating these classifications.

Author	Year	Country	Scoring criteria	Changes	Study type
Ghidey and Habte [29]	1983	Ethiopia	New	New	Prospective
Migliori et al. [30]	1992	Uganda	Migliori—revised from Ghidey and Habte	Focused towards PTB, added response to treatment as a criteria	Prospective
Madhi et al. [31]	1999	South Africa	Migliori	No change	Prospective
Salazar et al. [32]	2001	Peru	Migliori	Removed response to treatment. Created Peru criteria.	Prospective cohort

Marais et al. [26]	2006	South Africa	New	Symptom based approach	Prospective

World Health Organization [20]	1983	Multiple	New	New	New guidelines
Cundall [33]	1986	Kenya	1983 WHO guidelines	Modifies by adding family contact	Prospective
Stoltz et al. [34]	1990	South Africa	Modified 1983 WHO guidelines	No change	Prospective
Beyers et al. [35]	1994	South Africa	1983 WHO guidelines	No change	Prospective
Gie et al. [36]	1995	South Africa	Modified 1983 WHO guidelines	No change	Prospective
Schaaf et al. [37]	1995	South Africa	1983 WHO guidelines	No change	Prospective
Houwert et al. [38]	1998	South Africa	1994 WHO guidelines	No change	Prospective
Kiwanuka et al. [42]	2001	Malawi	1983 WHO guidelines	Modified by using only certain radiological findings or positive TST for probable TB	Prospective
Palme et al. [39]	2002	Ethiopia	Modified 1983 WHO guidelines	Required 2/6 criteria	Prospective case-control
Theart et al. [40]	2005	South Africa	Modified 1983 WHO guidelines	No change	Retrospective
Cohen et al. [41]	2008	UK	2006 WHO classification	No change	Retrospective

Osborne [43]	1995	Zambia	Lusaka's UTH Criteria	New	Review article
Jeena et al. [44]	1996	South Africa	Lusaka's UTH criteria	No change	Prospective

Ramachandran [45]	1968	India	New	New	Prospective and retrospective

**Table 3:** Studies evaluating and comparing multiple diagnostic systems.

Author	Year	Country	Findings
Hesseling et al. [21]	2002	South Africa	Analyzed 16 diagnostic systems, specifically looks at how systems have been adapted for HIV-infected and malnourished patients.
Edwards et al. [47]	2007	Congo	Analyzed 8 scoring systems, found correlation to be poor to moderate. Decision to initiate treatment for TB was dependent on scoring system used in 14% of children. Selection had a greater impact in HIV-infected patients.
Ahmed et al. [48]	2008	Bangladesh	Reviews previous scoring systems as well as Hesseling et al. [21] and Edwards et al. [47]
Raqib et al. [49]	2009	Bangladesh	Analyzed a new diagnostic test (ALS assay) detecting antibodies secreted from circulating MTB-specific plasma cells in comparison to the Kenneth Jones and WHO/Keith Edwards scoring criteria as well as clinical diagnosis.

**Table 4:** Studies attempting validation of diagnostic systems.

Author	Year	Country	Scoring criteria	Validation	Gold standard
Point-based scoring systems					

Mathur et al. [9]	1974	India	Kenneth Jones	Sens 73% (original criteria) Sens 95% (modified criteria)	Clinical diagnosis
Shah et al. [12]	1992	India	Kenneth Jones	Compared modified criteria to previous Kenneth Jones	Previous KJ
Mehnaz and Arif [13]	2005	Pakistan	Kenneth Jones	Retrospective analysis	Clinical control and response to treatment
Viani et al. [8]	2008	Mexico	Stegen-Toledo	Retrospective analysis	Clinical diagnosis

van Beekhuizen [16]	1998	Papua New Guinea	Keith Edwards	Sens 62%, spec 95%	Improvement on anti-TB treatment or positive CXR
Weismuller et al. [17]	2002	Malawi	WHO score chart (modified Keith Edwards)	Sens 61% for all types of TB; 54% for PTB and 73% for EPTB	Clinical diagnosis—differed by various hospitals
van Rheenen [18]	2002	Zambia	Keith Edwards	Sens 88%, spec 25%, PPV 55%, NPV 67%	Diagnostic algorithm
Narayan et al. [19]	2003	India	Keith Edwards	Sens 91%, spec 88%	Clinical diagnosis

Sant'Anna et al. [24]	2006	Brazil	Brazil Ministry of Health	Sens 89%, spec 86%	Culture positive and respiratory symptoms and/or CXR improved using exclusively anti-TB drugs
Sant'Anna et al. [25]	2004	Brazil	Brazil Ministry of Health	82% very likely, 16% possible, 2.4% unlikely	Clinical criteria and response to treatment
Pedrozo et al. [23]	2009	Brazil	Brazil Ministry of Health	Median score of TB positive groups higher than negative	Clinical criteria

Fourie et al. [27]	1998	Multiple	New	Analyzed by age and country group: sens 30–73%, spec 10–75%, PPV 50–82%	Positive radiologic or bacteriological data

Diagnostic classification					

Migliori et al. [30]	1992	Uganda	Migliori	Gastric aspirate: sens 96.8%, spec 92.2%, PPV 68.2%, NPV 99.4%. Response to treatment: sens 62.5%, 94.1%, PPV 57.7%, NPV 95.1%	Original Ghidey and Habte criteria
Salazar et al. [32]	2001	Peru	Migliori	Sens 92% (Migliori) versus 80% (Peru). 3/3 Peru criteria had 73% PPV	Migliori criteria (without RTT)

Marais et al. [26]	2006	South Africa	New	Children ≥3 and HIV uninfected: sens 82.3%, spec 90.2%, PPV 82.3%. Children <3 and HIV uninfected: sens 51.8%, spec 92.5%, PPV 90.1%. HIV infected: sens 56.2%, spec 61.8%, PPV 61.9%	Clinical criteria

Houwert et al. [38]	1998	South Africa	WHO provisional guidelines (1994)	PPV of all 3 criteria when present simultaneously: 63%	WHO diagnostic categories from 1994 used as the gold standard

Sens: sensitivity; spec: specificity; PTB: pulmonary tuberculosis; EPTB: extrapulmonary tuberculosis; PPV: positive predictive value; NPV: negative predictive value.

**Table 5:** Studies focusing primarily on pulmonary tuberculosis.

Author	Year	Country	Scoring system	Percent also with EPTB	Validation
Shah et al. [12]	1992	India	Modified Kenneth Jones	Looked at “primary complex” (just pulmonary) versus “progressive primary complex” (pulmonary plus LAD)	Not analyzed, just used in inclusion criteria

Migliori et al. [30]	1992	Uganda	Migliori	All pulmonary	Gastric aspirate: sens 96.8%, spec 92.2%, PPV 68.2%, NPV 99.4%. Response to treatment: sens 62.5%, 94.1%, PPV 57.7%, NPV 95.1%

Beyers et al. [35]	1994	South Africa	Modified 1883 WHO criteria	All pulmonary—excluded extrapulmonary tuberculosis without lung involvement	Not evaluated

Salazar et al. [32]	2001	Peru	Migliori	All pts had PTB, 21/135 had EPTB as well: lymphadenopathy, intestinal-intraperitoneal TB, intra-abdominal lymphadenopathy, miliary disease, meningitis, and optic involvement. 3 with EPTB did not meet criteria for PTB	Sens 92% (Migliori) versus 80% (Peru). 3/3 Peru criteria had 73% PPV

Sant'Anna et al. [25]	2004	Brazil	Brazil Ministry of Health	82% very likely, 16% possible, 2.4% unlikely	All pulmonary plus 5 pts with assoc extrapulmonary TB

Sant'Anna et al. [24]	2006	Brazil	Brazil Ministry of Health	Cut off ≥40: sens 58% and spec 98% but missed 42% of confirmed PTB. Cut off ≥30: sens 89% and spec 86%	Pulmonary

Oberhelmen et al. [14]	2006	Peru	Stegen-Toledo	Not analyzed, just used in inclusion criteria	Pulmonary

Marais et al. [26]	2006	South Africa	New	Children ≥3 and HIV uninfected: sens 82.3%, spec 90.2%, PPV 82.3%. Children <3 and HIV uninfected: sens 51.8%, spec 92.5%, PPV 90.1%. HIV infected: sens 56.2%, spec 61.8%, PPV 61.9%	Focused on pulmonary TB only

Viani et al. [8]	2008	Mexico	Stegen-Toledo	Looked retrospectively: 10/13 highly probable, 2/13 probable, 1/13 suspicious	100% pulmonary, 54% also had disseminated

Pedrozo et al. [23]	2009	Brazil	Brazil Ministry of Health	Analyzed scoring system by looking at median scores of various groups: median score of 3a and 3b sig. higher than 1 and 2, median score also was higher than the cut off of 30	Pulmonary only

Sens: sensitivity; spec: specificity; PTB: pulmonary tuberculosis; EPTB: extrapulmonary tuberculosis; PPV: positive predictive value; NPV: negative predictive value.

**Table 6:** Studies that specified how many patients were coinfected with HIV.

Author	Year	Country	Total patients	Percent HIV positive	Findings
Madhi et al. [31]	1999	South Africa	130	40%	Did not attempt to validate scoring criteria

Kiwanuka et al. [42]	2001	Malawi	110	71% (of 102 tested)	Did not attempt to validate scoring criteria

Palme et al. [39]	2002	Ethiopia	517	11.2%	Did not attempt to validate scoring criteria

van Rheenen [18]	2002	Zambia	147	30%	Keith Edwards scoring system: sensitivity 88% and specificity 25% in this study. Most of the children with a false positive score were malnourished (48%) or had AIDS (31%)

Marais et al. [26]	2006	South Africa	428	8.8%	Sensitivity, specificity, and PPV all decreased significantly when HIV infected children included

Edwards et al. [47]	2007	Democratic Republic of Congo	91	46%	Out of 8 scoring systems analyzed, 3/8 systems did not recommend treatment in 14% of HIV-infected children compared to 2% of noninfected children. Mean score tended to be higher for HIV-infected children, but only significant for Edwards score

Viani et al. [8]	2008	Mexico	13	100%	Applied Stegen-Toledo criteria retrospectively but without culture results: 77% had highly probable TB, 15% probable, and 8% suspicion of TB

Pedrozo et al. [23]	2009	Brazil	239	5%	Analyzed scoring system by looking at median scores of various groups: median score of 3a (TB+, HIV−) and 3b (TB+, HIV+) sig. higher than TB negative groups, median score of TB+ groups also was higher than the cutoff of 30

PPV: positive predictive value.
